# Comparative Analysis of Differentially Expressed miRNAs and their Downstream mRNAs in Ovarian Cancer and its Associated Endometriosis

**DOI:** 10.4172/1948-5956.1000359

**Published:** 2015

**Authors:** Richard Licheng Wu, Shadan Ali, Sudeshna Bandyopadhyay, Baraa Alosh, Kinda Hayek, MHD Fayez Daaboul, Ira Winer, Fazlul H Sarkar, Rouba Ali-Fehmi

**Affiliations:** 1 Department of Pathology, Karmanos Cancer Institute, Wayne State University School of Medicine, Detroit, Michigan, USA; 2 Department of Oncology, Karmanos Cancer Institute, Wayne State University School of Medicine, Detroit, Michigan, USA; 3 Department of obstetrics and Gynecology, Karmanos Cancer Institute, Wayne State University School of Medicine, Detroit, Michigan, USA

**Keywords:** Ovarian cancer, Endometriosis, miRNA, PTEN, NF-κB, FFPE

## Abstract

**Objective:**

There is an increased risk of developing ovarian cancer (OC) in patients with endometriosis. Hence, development of new biomarkers may provide a positive clinical outcome for early detection. MicroRNAs (miRNAs) are small non-coding RNAs that play an important role in biological and pathological process and are currently used as diagnostic and prognostic markers in various cancers. In the current study, we assessed the differential expression of miRNAs from 19 paired ovarian cancer and its associated endometriosis tissue samples. In addition we also analyzed the downstream targets of those miRNAs.

**Methods:**

Nineteen paired cases of ovarian cancer and endometriosis foci were identified by a gynecologic pathologist and macro-dissected. The total RNAs were extracted and subjected to comprehensive miRNA profiling from the pooled samples of these two different entities using microarray analysis. Later, the abnormal expressions of few selected miRNAs were validated in individual cases by quantitative real-time PCR (qRT-PCR). Ingenuity pathway analysis revealed target mRNAs which were validated by qRT-PCR.

**Results:**

The miRNA profiling identified deregulation of greater than 1156 miRNAs in OC, of which the top seven were further validated by qRT-PCR. The expression of *miR-1*, *miR-133a*, and *miR-451* were reduced significantly (p<0.0001) in the OC patients compared to its associated endometriosis. In contrast, the expression of *miR-141*, *miR-200a*, *miR-200c*, and *miR-3613* were elevated significantly (p<0.05) in most of the OC patients. Furthermore, among the downstream mRNAs of these miRNAs, the level of PTEN expression was significantly (p<0.05) reduced in OC compared to endometriosis while no significant difference was observed in NF-κB expression.

**Conclusion:**

The expression of miRNAs and mRNAs in OC were significantly different compared to its concurrent endometriosis. These differential expressed miRNAs may serve as potential diagnostic and prognostic biomarkers for OC associated with endometriosis.

## Introduction

Ovarian cancer (OC) is the leading cause of cancer associated death among gynecologic malignancies [1]. Recently, microRNAs (miRNAs) have drawn the attention of researchers due to its significant roles in the tumorigenesis and progression, in addition to their essential role in normal mammalian development [2]. Although numerous studies have been conducted to elucidate the mechanisms of carcinogenesis in OC, only a few are exploring the role of miRNAs in the development and characterization of OC.

miRNAs are a class of small, non-coding RNAs, which are involved in multiple normal biological pathways including cell differentiation, apoptosis, proliferation and metabolism by splicing and regulating mRNAs [3]. Experimental evidence supports the potential roles of miRNAs in cancer pathogenesis through their function as oncogenes or tumor suppressive genes in multiple cancers [4–7]. Current studies have shown that miRNAs are remarkably stable, remain largely intact and can be detected not only in plasma but also in fresh and frozen tissue and in formalin-fixed paraffin-embedded (FFPE) tissues, suggesting the possibility of utilizing these as a new class of biomarkers for diagnosis and prognosis in cancer [4,8].

Endometriosis is a common benign gynecological process defined pathologically as presence of endometrial glands and stroma outside of the uterus. It may cause pelvic pain, intestinal disorders and infertility [9]. Many reports have identified endometriosis as a significant risk factor for OC [1,10–12]. We collected nineteen ovarian tumor cases from patients with endometriosis and concurrent invasive OC. The presence of these two lesions provides an opportunity to study their similarities and differences at the molecular level.

In our study, a comprehensive miRNA profiling from the OC and its concurrent endometriosis were compared to identify the key miRNAs that distinguish these two entities. Some of these miRNAs were further validated individually in each patient by direct comparison between paired endometriosis and OC samples using qRT-PCR. Furthermore, mRNA targets of these differentially expressed miRNAs were identified and validated individually using qRT-PCR. Identification of downstream targets of these miRNA may ultimately provide therapeutic targets in the future.

## Materials and Methods

### Tissue collection

We identified 49 cases of OC with concurrent endometriosis from the database of the pathology department of Wayne State University diagnosed between 1984–2012. After obtaining approval from the institutional review board, a retrospective chart review of the patient data was performed. Slides were reviewed by a gynecologic pathologist. Nineteen cases were confirmed to have OC with associated foci of endometriosis. Of these cases, five were endometrioid, eight serous and one clear cell carcinoma and five mixed carcinoma.

### RNA isolation

Total RNA was extracted using RNeasy Kit (Qiagen, Valencia, CA,) from formalin-fixed paraffin embedded (FFPE) tissue sections according to the manufacturer’s protocol with some modifications. The endometriosis foci and OC foci from each case were selected under microscope and macro-dissected precisely. Based on the size of the foci, 4–10 freshly cut tissue sections of 10 μm thick were placed in micro tubes along with 1 ml xylene. After vigorous shaking, samples were centrifuged for 2 min at room temperature. About 1 ml of ethanol was added and centrifuged for 2 min. The resultant pellet was resuspended in 240 μl of buffer PKD along with 10 μl of proteinase K and incubated at 55°C for 15 min, and then at 80°C for 15 min. The lower uncolored phase was transferred into a micro tube and centrifuged for 15 min at 20,000 g. The supernatant was next transferred to a new tube along with DNase I stock solution and DNase booster buffer and incubated at room temperature for 15 minutes. Approximately 500 μl of buffer RBC was then added and mixed with 1200 μl of ethanol and was subsequently applied to the RNeasy MinElute spin column and centrifuged at 8,000 g for 15 sec. The bound RNA was washed with RPE buffer solution twice to remove impurities and eluted with RNase free water. RNA was quantified and its purity evaluated by the absorption ratio at 260/280 nm using NanoDrop 2000 (Thermo Scientific, Pittsburgh, PA,). The ratio of 260/280 varied from 1.8–2.1.

### MicroRNA profiling

Purified total RNAs were pooled separately from aliquots of 19 benign endometriosis or OC samples and submitted these two pooled RNAs (endometriosis and OC) for analysis using a service provider (LC Sciences, Houston, TX) for comprehensive miRNA microarray profiling. The miRNA profiling was performed by miRBase version 19 (LC Sciences). The data were normalized using selected housekeeping genes. Network analysis was accomplished with the web-based bioinformatics tool, Ingenuity pathway analysis software (Ingenuity Systems, Redwood City, CA) to identify the target mRNAs from these differentially expressed miRNAs.

### Real-time reverse transcriptase-PCR of miRNAs (qRT–PCR)

Expression of the top seven differentially expressed miRNAs by microarray profiling was validated using quantitative real-time PCR (qRT-PCR). In brief, total RNA (10 ng) was reverse transcribed using specific miRNA primers and the TaqMan miRNA Reverse Transcription Kit (Life Technologies, Grand Island, NY). The resulting cDNA was used as input in a qRT-PCR using the miRNA specific probes mix and the TaqMan Universal PCR Master Mixture according to manufacturer’s protocol (Life Technologies). All reactions were performed in triplicate. The relative expression of miRNAs was analyzed using the **C**_t_ method and was normalized by RNU48 expression.

### Real-time reverse transcriptase-PCR of mRNAs (qRT–PCR)

The mRNA level of NF-κB and PTEN was determined by qRT-PCR using High Capacity RNA-to-cDNA kit (Life Technologies, Grand Island, NY). In brief, total RNA (500 ng) was reverse transcribed using High Capacity RNA-to-cDNA kit. The resulting cDNA was used as input in a qRT-PCR using NF-κB and PTEN primers together with the SYBR Green Master mix according to manufacturer’s protocol (Life Technologies). All reactions were performed in triplicate. The relative expression of mRNAs was analyzed using the **C**_t_ method and was normalized by GAPDH expression.

### Statistical analysis

A paired *t*-test was used to determine the significance of differential miRNA expression between endometriosis and ovarian cancer obtained by qRT-PCR. The significance of PTEN and NF-κB mRNA level in individual cases was analyzed by student t-test. A level of *P* value < 0.05 was regarded as statistically significant and *P* value < 0.01 was regarded as statistically very significant.

## Results

### Patients clinicopathologic characteristics

**Figure 1:**
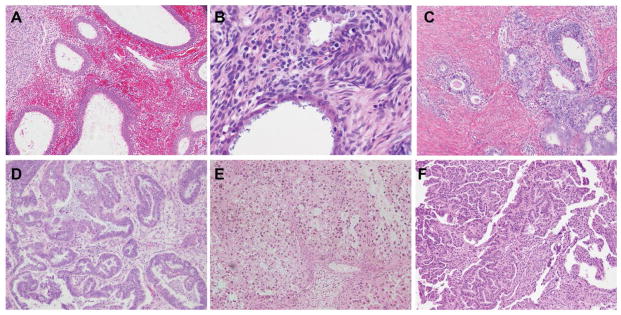
Representative foci of endometriosis and ovarian cancers (OC) for miRNA array assay. Endometriosis (A, B, C), Endometrioid carcinoma (D), Clear cell carcinoma (E), Serous carcinoma (F).

The study cohort included nineteen cases of OC with concomitant endometriosis. All cases were morphologically confirmed by a gynecologic pathologist. The OC cases included one clear cell carcinomas, five endometrioid carcinomas, eight serous carcinomas and five mixed carcinomas (Figure 1). The characteristics of the patients are described in Table 1. The study group comprised of 15 Caucasians, 3 African Americans and 1 other race with age range 25–70 years old (median 53 years old) (Table 1). Six patients (6/19) had advanced stage disease (stage III and IV). Of these five were in the serous carcinoma group (5/8) and one was in the mixed carcinoma group (1/5) with serous component. Lymph node metastasis was present in 3 of serous carcinoma cases (3/8) (Table 2). Endometriosis foci were present at different locations in all cases. Eleven cases (11/19) demonstrated endometriosis and OC in the same ovary and five cases (5/19) showed endometriosis and OC present in opposite ovaries. Two cases had endometriosis present on the serosal surface of uterus and one in the fallopian tube (Table 3).

### Expression profiling of miRNAs

Aliquots of RNA extract from FFPE tissue blocks of ovarian cancer and benign endometriosis (approximately 200 ng each) were pooled separately. Microarray technology has permitted a comprehensive analysis of all miRNAs that are differentially expressed in OC and endometriosis. The Raw data of the comprehensive miRNA expression profiling has been deposited in NCBI’s Gene Expression Omnibus (GEO) which is a public functional genomics data repository and are accessible through GEO Series accession number GSE71477.

**Figure 2:**
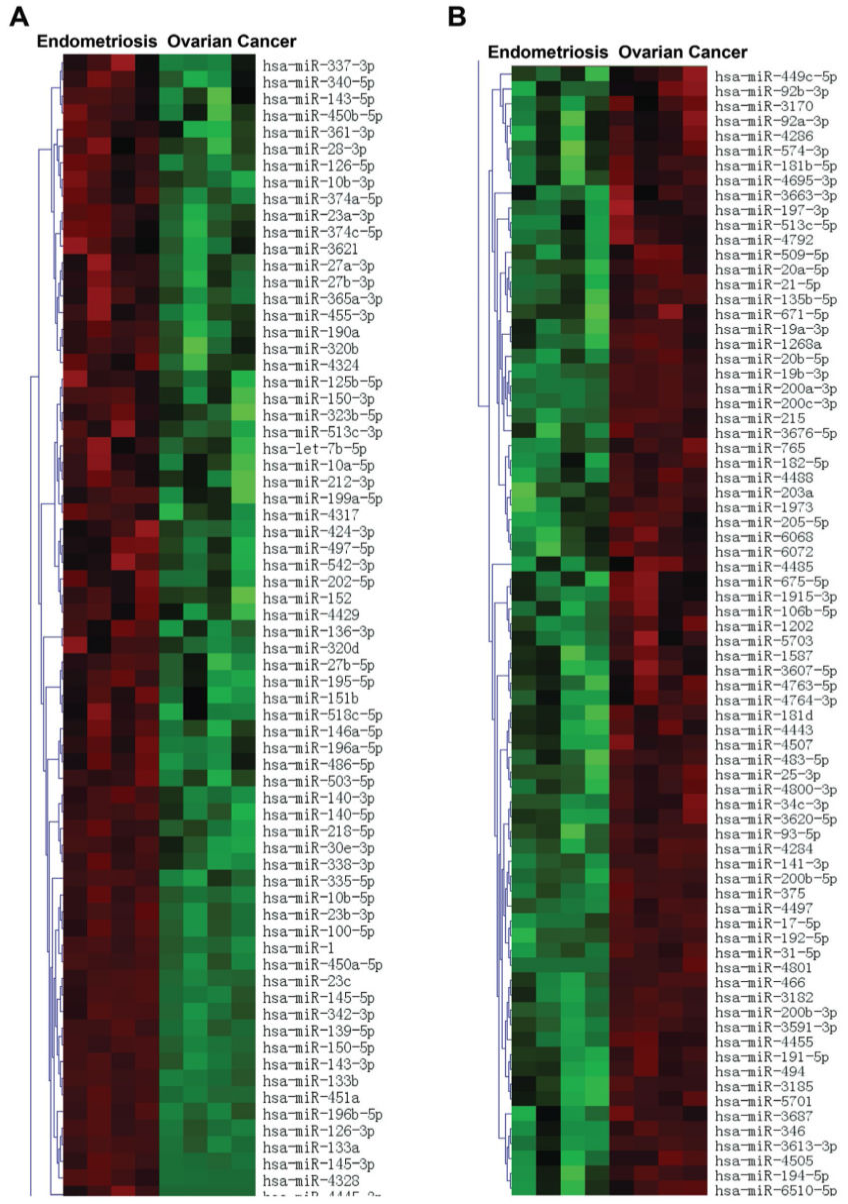
Heatmap of clustering miRNA microarray data with columns arranged according to hierarchical clustering method. miRNA groups that are increased in endometriosis compared to ovarian cancer (A). miRNA groups that are reduced in endometriosis compared to ovarian cancer (B).

Expression profiling revealed 1156 miRNAs differentially expressed in subjects with ovarian cancer but not endometriosis. The 150 interesting miRNAs, that are differentially expressed in the ovarian cancer compared to endometriosis significantly (p < 0.05) with signal intensity larger than 500.00, are presented in Figure 2. Among these significantly differentially expressed miRNAs, we chose three tumor suppressor and four oncogenic miRNAs based on log ratio ~ 2.00. These included *miR-1*, *miR-133a*, *miR-451*, *miR-141*, *miR-3613*, *miR-200a*, and *miR-200c* which were further validated individually using qRT-PCR as presented below.

### qRT-PCR of up- and down-regulated selected miRNAs

**Figure 3:**
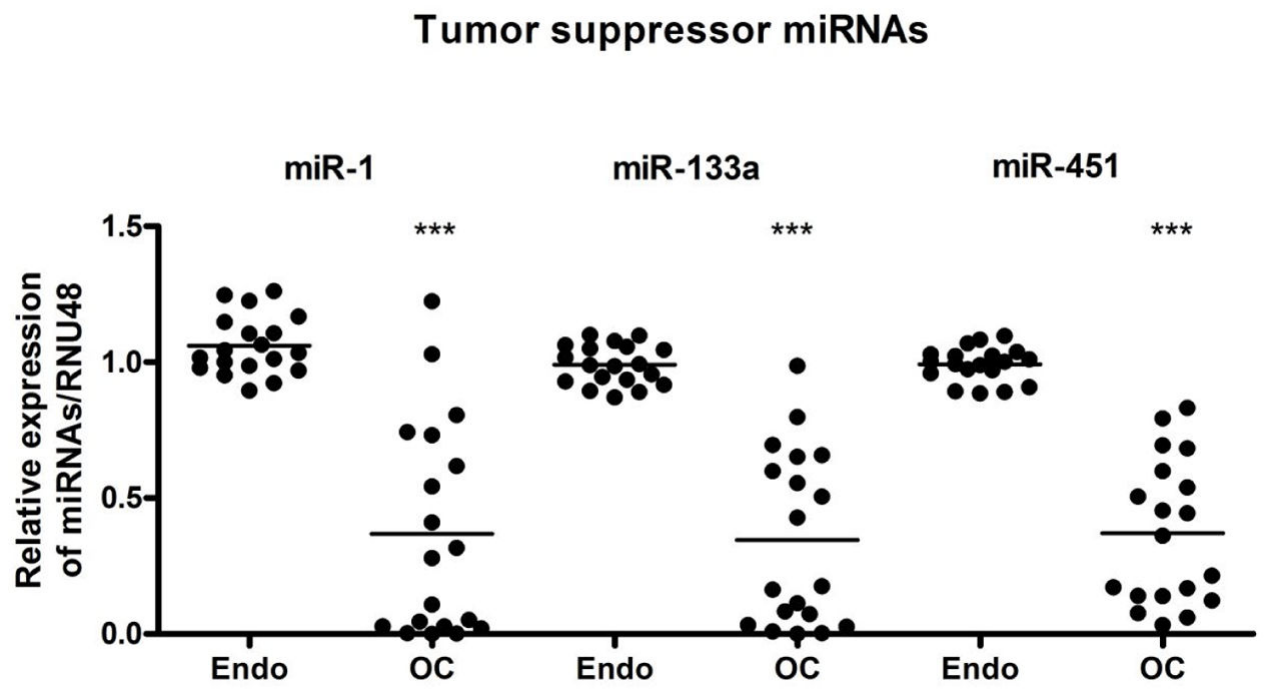
Comparative expression analysis of *miR-1*, *miR-133a* and *miR-451* in 19 paired samples of FFPE blocks of endometriosis (Endo) and ovarian cancer (OC) tissue samples individually using qRT-PCR. There was a significant down regulation of all three miRNAs tested in OC compared to respective Endo. *** represent comparison between Endo and OC using *t* test and the *p value* was found to be < 0.0001. RNU48 was used as loading control.

**Figure 4:**
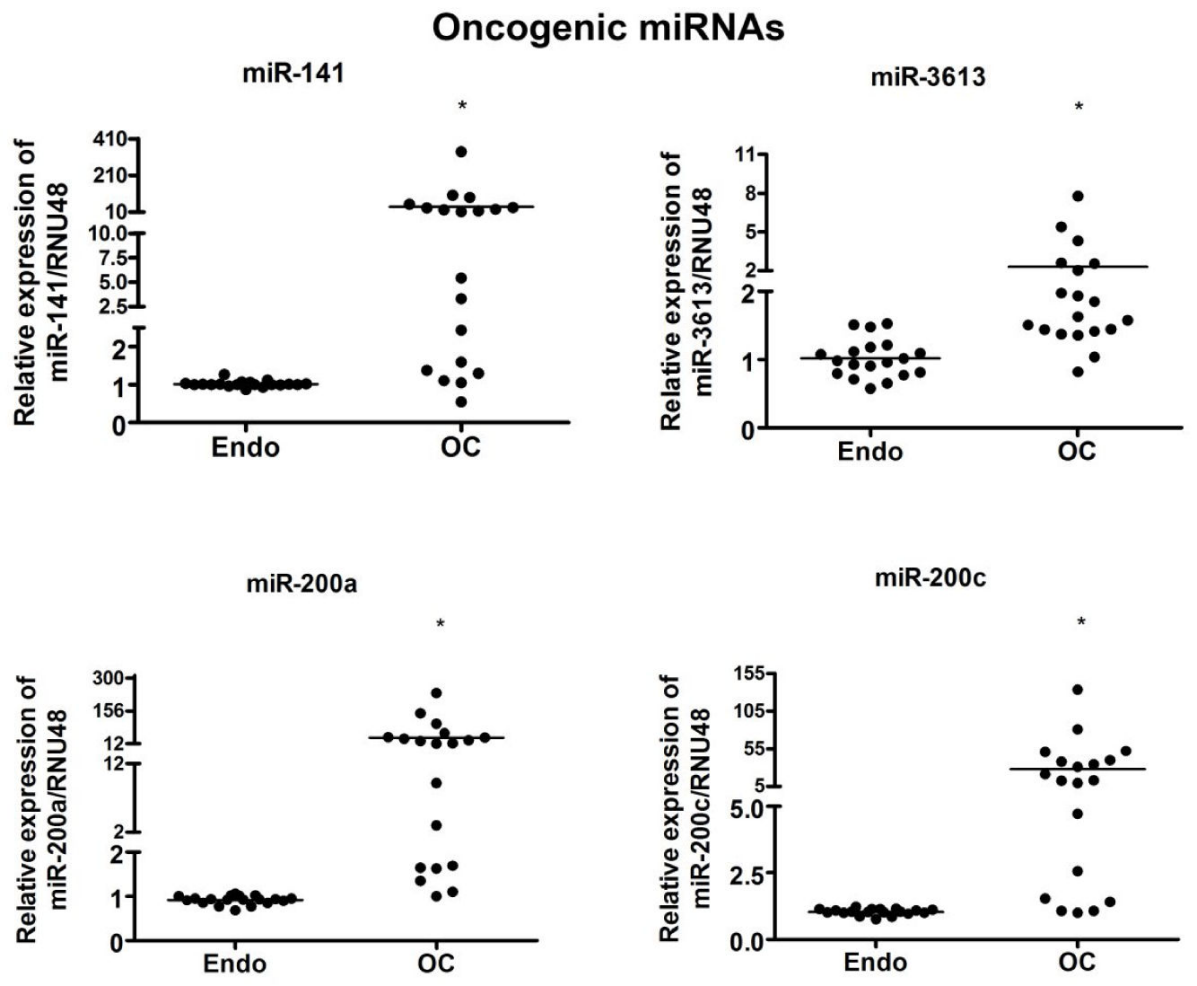
Comparative expression analysis of *miR-141*, *miR-3613*, *miR-200a* and *miR-200c* in 19 paired samples of FFPE blocks of endometriosis (Endo) and ovarian cancer (OC) tissue samples individually using qRT-PCR. There was a significant up regulation of all four miRNAs tested in OC (ovarian cancer) compared to respective Endo (endometriosis) as assessed by qRT-PCR. * represent comparison between Endo and OC using *t* test and the *p value* was found to be < 0.05. RNU48 was used as loading control.

The miRNA expressions of seven miRNAs were further validated in nineteen paired samples individually based on the miRNA profiling data by qRT-PCR. The analysis was carried out in triplicate and in parallel to prevent batch effects. The relative differential expression of the selected miRNAs in ovarian cancer was demonstrated by setting the expression level of endometriosis at 1.0. The miRNA expression analysis of three down-regulated miRNAs, *miR-1*, *miR-133a*, and *miR451* showed significant reduction in ovarian tumors compared to their paired benign endometriosis (p < 0.001), as shown in Figure 3. In addition, the expression of the four up-regulated miRNAs, *miR-200a*, *miR-200c*, *miR-141*, *miR-3613*, showed significant up-regulation in ovarian tumors compared to their benign endometriosis (p < 0.05) (Figure 4).

### Ingenuity pathway analysis (IPA) of expressed miRNAs

**Figure 5:**
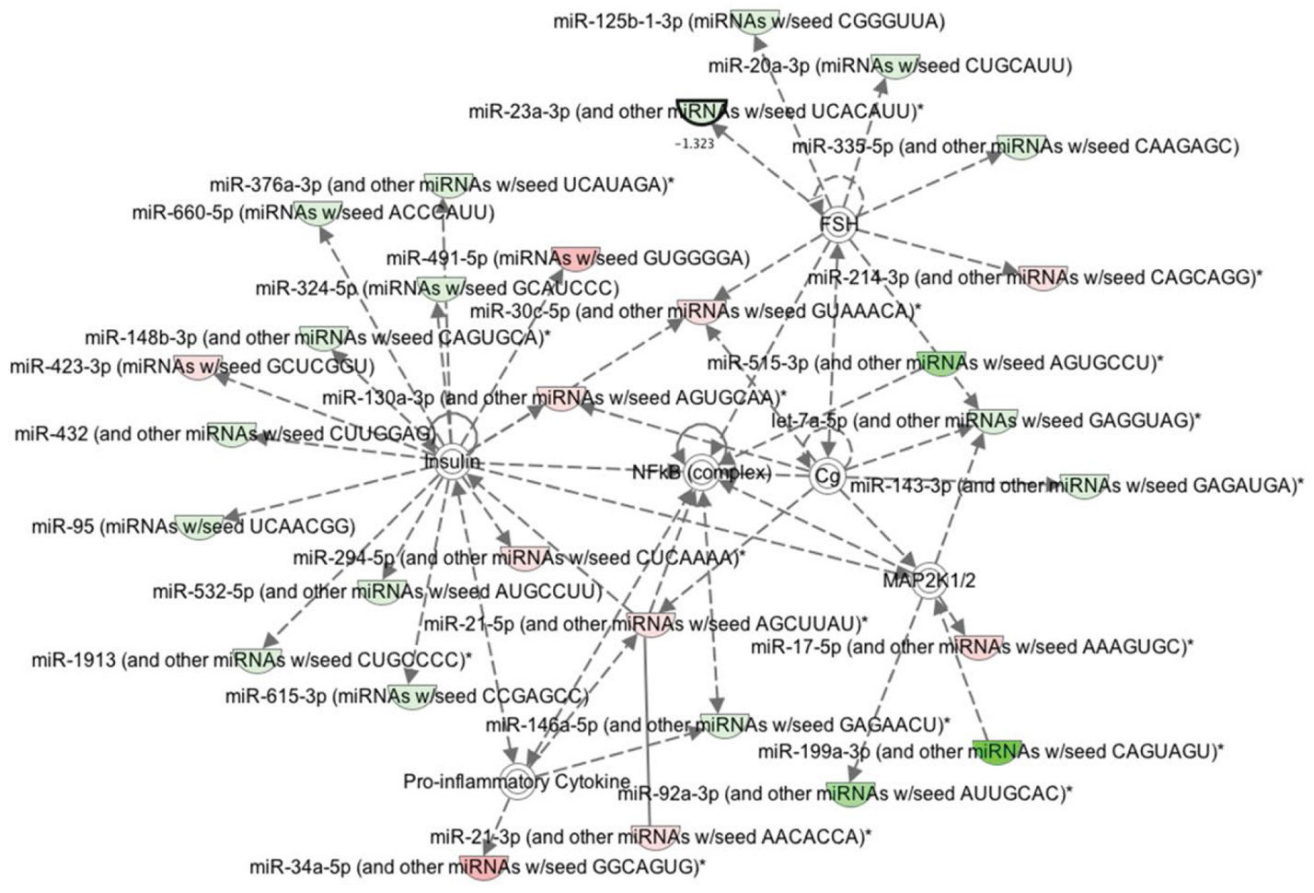
Ingenuity pathway analysis showing up (green) and down-regulation (red) of miRNAs involved in ovarian cancer tumor samples when compared to endometriosis samples. Target genes are also represented, such as Insulin, NF-κB, and MAP2K 1/2 pathways.

In order to comprehensively describe the pathways involved and their target genes, ingenuity modeling of the expression profiling of miRNAs was executed. Interestingly, among the different hubs that were activated in IPA derived networks, NF-κB and its related network mediator PTEN were found to be activated in OC samples as depicted in Figure 5. Hence, we investigated the mRNA expression level of both NF-κB and PTEN using qRT-PCR in the same paired samples.

### The mRNA expression level of NF-κB and PTEN in ovarian cancer measured by qRT-PCR

**Figure 6:**
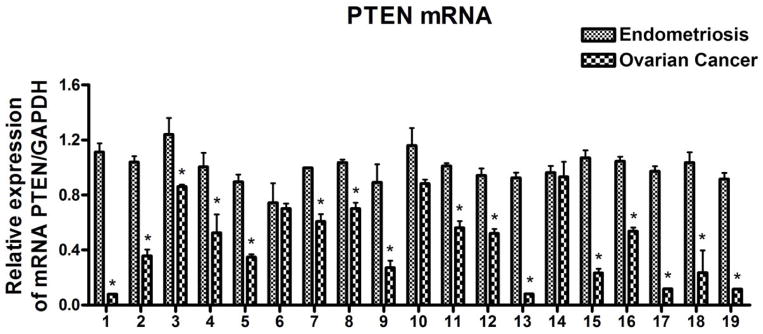
Comparative expression analysis of PTEN in 19 paired samples of FFPE blocks of endometriosis and ovarian cancer tissue samples individually using qRT-PCR. PTEN mRNA was significantly reduced in ovarian cancer compared to endometriosis as assessed by qRT-PCR. * represent comparison between Endometriosis and ovarian cancer using *t* test and the *p value* was found to be < 0.05. GAPDH was used as loading control.

In order to further verify the target genes identified by IPA among ovarian cancer and endometriosis, we investigated the PTEN and NF-κB expression in paired samples of OC and endometriosis. The experiments were carried out in triplicate and in parallel to prevent batch effects. The mRNA values of PTEN and NF-κB were normalized using GAPDH mRNA. The relative changes in mRNA expression of PTEN and NF-κB of OC were first demonstrated by setting endometriosis level at 1.0. We observed significantly reduced PTEN expression in sixteen individual OC cases (16/19) when compared to endometriosis (p < 0.05) as presented in Figure 6. However, the expression level of NF-κB was not significantly altered between OC and endometriosis foci, which suggested that the activation of the NF-κB pathway through phosphorylation directly or indirectly altered by miRNAs without changes in its expression level.

## Discussion

Endometriosis is a common condition among reproductive-aged women affecting approximately 5% to 10% of women during their lifetime [13]. There is an increased risk of developing OC in 2.0–17.0% of patients with endometriosis [14,15]. Little is known about the biomarkers that can assess the similarities and differences between OC and its associated endometriosis. Identification of the miRNAs that distinguish OC from benign endometriosis, such as *miR-1*, *miR-133a* and *miR-451* may serve as potential biomarkers in patients with endometriosis for screening for OC.

A recent study by Cao Q, et al. observed a significant increase in *miR-200a*, *miR-200b*, *miR-200c* and *miR-141* in ovarian cancer [16], which have been consistently reported in other profiling studies [17]. These comparisons were performed between normal ovarian epithelial cells and the epithelial ovarian cancer cells [16]. In the present study, comparable differences among the miRNAs especially *miR-200a*, *miR-200c* and *miR-141* were observed between the OC and its concurrent endometriosis. These miRNAs belong to the same *miR-200* family. The function of *miR-200* family members and their interactions with other molecular components are the key to dissect the possible mechanisms that suggested the invasive features of OC. The most important targets of the *miR-200* family are two E-box binding transcription factors, ZEB1 and ZEB2, which result in the inhibition of transcription of some epithelial polarity associated proteins, such as E-cadherin [18]. The dysregulation of the epithelial polarity and the differentiation are involved in the epithelial to mesenchymal transition (EMT) [18]. The E-cadherin-mediated intercellular association disappears during the tumor progression in majority of the carcinomas and the expression of N-cadherin occurs concurrently [19]. However, these expression patterns are highly complex and not consistent in the normal and neoplastic ovary. The E-cadherin expression is decreased in some primary ovarian carcinoma and is re-expressed in a greater amount in ovarian carcinoma effusions, suggesting incomplete transformation to EMT [19]. Because EMT plays an important role in the tumor metastases and progression, the upregulated *miR-200* family members may transform tumor cells from indolent status to more aggressive and invasive phenotype. Elgaaen et al. reported over-expression of *miR-200* family and under expression of ZEB1, ZEB2 and vimentin in high-grade serous ovarian carcinoma (HGSC) and clear cell ovarian carcinoma (COC) which were further confirmed by Ingenuity pathway analysis (IPA) documenting ZEB1 and ZEB2 as the most under expressed mRNAs in HGSC compared to ovarian surface epithelium [20]. Therefore, these miRNAs (*miR-200a*, *miR-200c* and *miR-141*) may serve as biomarkers for OC during the emergence of invasive OC from normal reactive ovarian epithelial tissues or a benign entity, such as endometriosis. However, the role of EMT and MET related events associated with ZEB1, ZEB2, E-cadherin, vimentin and *miR-200* family is highly complex, which certainly requires further investigation in the context of endometriosis and ovarian cancer.

*MiR-1* is down regulated in many malignant tumors, such as non-small cell lung cancer, glioblastoma etc [21,22]. It is one of the evolutionarily conserved miRNAs that share common expression in the muscle cells from *C. elegans* to human. It has also been shown to be involved in the angiogenesis during the muscular development of zebrafish [23]. Godlewski showed that the re-expression of *miR-1* in glioblastoma cells inhibited *in vivo* growth, neo-vascularization, and invasiveness [24]. We have demonstrated that *miR-1* expression was significantly down regulated in the OC compared to the benign endometriosis. It has been reduced by more than 1000 times in some of the OC samples. However, the exact mechanisms and its downstream targets by which it suppresses the tumorigenesis need to be further investigated.

*MiR-451* was reported to be frequently down regulated in several types of tumors, [25] such as gastric cancer [26], lung cancer [27], glioma [28,29] and breast cancer [29]. Several targets of *miR-451*, such as calcium binding protein 39 (CAB39) [29], Ras-related protein 14 (RAB14) [27] which are related to the cell cycling and proliferation, have been identified. We observed that *miR-451* was significantly dysregulated in the ovarian carcinoma. However, little is known about the *miR-451* function and its mechanisms regarding the invasive behavior of OC and needs to be further studied.

The down regulation of *miR-133a* has been demonstrated in different types of carcinoma. Ectopic expression of *miR-133a* inhibited colorectal carcinoma (CRC) cell proliferation and migration. Over-expression of *miR-133* suppressed the CRC proliferation and metastasis *in vivo* [30,31] by targeting the 3′ untranslated region (3′UTR) of *LIM and SH3 protein 1* (*LASP1*) mRNA, that are CRC associated proteins [31]. The key components of MAPK pathway, such as ERK and MEK, are modulated by *miR-133a* in CRC [32]. Guo et al. have shown that *miR-133a* suppressed the OC proliferation by targeting the insulin like growth factor receptor [33]. The ectopic expression of *miR-133a* suppressed the OC cell proliferation and colony formation significantly by inducing the G1- phase cell cycle arrest [33]. In addition, over expression of *miR-133a* suppressed *in vivo* tumor growth in nude mice [33]. The significantly suppressed *miR-133a* in OC was observed in our study when compared to the benign endometriosis indicating the important role of *miR-133a* and it’s targeting mRNA in the formation of the ovarian carcinoma. This observation suggests that *miR-133a* may be used as biomarker and therapeutic target for diagnosis and therapy of OC.

Annunziata et al. found that NF-κB pathway is over activated in aggressive ovarian cancers and that some target matrix involved in the NF-κB pathway is associated with poor prognosis [34]. Therefore, we measured NF-κB mRNA levels between endometriosis and associated OC. However, in our study, the NF-κB mRNA expression level did not show any significant differences in the aggressive OC compared to coexisting benign endometriosis from same patient. Although some of these differentially expressed oncogenic miRNAs in OC target NF-κB mRNA, its mRNA level may not change due to the complexity of its interactive loops involved in these regulatory miRNAs. Therefore, the identification of the key miRNAs among these differentially expressed miRNAs, which leads to the formation of the malignant OC, may play a role in future studies concerning targeted therapy.

Dinulescu et al. demonstrated the direct link between endometriosis and endometrioid carcinoma through transgenic mouse model. Moreover, the tissue specific expression of mutant K-ras caused endometriosis while expression of active K-ras in combination with PTEN inactivation caused metastatic endometrioid carcinoma that resembled human OC [35]. In the present study, we observed a significant decrease in PTEN mRNA expression level along with tumor suppressor miRNA (1, 133a, 451) in OC compared to endometriosis, which has been verified by real time PCR in most of the individual cases. Although the observation of PTEN expression level reduction is associated with transformation of benign endometriosis to malignant ovarian cancer in mouse model [35], the molecular stepwise progression of such transformation is not available.

Significant differences between OC and its associated endometriosis were observed at the level of miRNAs transcription. Both tumor suppressor miRNAs (1, 133a, 451) and oncogenic miRNAs (141, 200a, 200c, 3613) are possible molecules that distinguish ovarian cancer from its associated endometriosis. These miRNAs may be further studied to identify their role as possible mediated molecules in the development of ovarian cancer in patients with endometriosis. Identification and validation of downstream mRNA targets of these miRNAs, not only assists in the understanding of potential mechanisms involved in OC carcinogenesis, but also may lay the groundwork for new therapeutic targets in the future.

## Figures and Tables

**Table 1:** Demographic characteristics of patients with coexisting endometriosis and ovarian cancer.

Histologic type	Cases (N)	Age (Y) Median (Range)	CA	Race AA	Other
Clear cell carcinoma	1	64	1	0	0
Endometroid carcinoma	5	63(50–70)	3	1	1
Serous carcinoma	8	57(32–66)	6	2	0
Mixed type	5	58(25–57)	5	0	0
Total	19	53(25–70)	15	3	1

CA=Caucasian AA=African American

**Table 2:** Pathologic characteristics of ovarian cancer with coexisting endometriosis.

Histologic type	Cases(N)	Grade	Stage	Positive LN
Clear cell carcinoma	1	3	2	0/1
Endometroid carcinoma	5	1–2	1	0/5
Serous carcinoma	8	3	1–4	3/8
Mixed type	5	3	1–4	0/5
Total	19	1–3	1–4	3/19

LN: lymph node

**Table 3:** The site of endometriosis foci with its coexisted ovarian cancer on same patient.

Histologic type	Cases (N)	Ovary (S)*	Ovary (O)*	Endometriosis Serosa (T)*	Serosa (U)*
Clear cell carcinoma	1	0	0	0	1
Endometroid carcinoma	5	2	2	1	0
Serous carcinoma	8	4	3	0	1
Mixed type	5	5	0	0	0
Total	19	11	5	1	2

* Ovary (S): endometriosis existed with ovarian cancer at same site of ovary, Ovary (O): endometriosis and ovarian cancer present at different site of ovary, Serosa (T): endometriosis present on surface of the fallopian tube, Serosa (U): endometriosis present at surface of uterus

## References

[1] Brinton LA, Gridley G, Persson I, Baron J, Bergqvist A (1997). Cancer risk after a hospital discharge diagnosis of endometriosis. Am J Obstet Gynecol.

[2] Ali S, Almhanna K, Chen W, Philip PA, Sarkar FH (2010). Differentially expressed miRNAs in the plasma may provide a molecular signature for aggressive pancreatic cancer. Am J Transl Res.

[3] Zisoulis DG, Kai ZS, Chang RK, Pasquinelli AE (2012). Autoregulation of microRNA biogenesis by let-7 and Argonaute. Nature.

[4] Ali S, Saleh H, Sethi S, Sarkar FH, Philip PA (2012). MicroRNA profiling of diagnostic needle aspirates from patients with pancreatic cancer. Br J Cancer.

[5] Bonfrate L, Altomare DF, Di Lena M, Travaglio E, Rotelli MT (2013). MicroRNA in colorectal cancer: new perspectives for diagnosis, prognosis and treatment. J Gastrointestin Liver Dis.

[6] Gailhouste L, Gomez-Santos L, Ochiya T (2013). Potential applications of miRNAs as diagnostic and prognostic markers in liver cancer. Front Biosci (Landmark Ed).

[7] Melo SA, Esteller M (2011). Dysregulation of microRNAs in cancer: playing with fire. FEBS Lett.

[8] Lam EW, Shah K, Brosens JJ (2012). The diversity of sex steroid action: the role of micro-RNAs and FOXO transcription factors in cycling endometrium and cancer. J Endocrinol.

[9] Vlahos NF, Kalampokas T, Fotiou S (2010). Endometriosis and ovarian cancer: a review. Gynecol Endocrinol.

[10] Siufi Neto J, Kho RM, Siufi DF, Baracat EC, Anderson KS (2014). Cellular, histologic, and molecular changes associated with endometriosis and ovarian cancer. J Minim Invasive Gynecol.

[11] Aris A (2010). Endometriosis-associated ovarian cancer: A ten-year cohort study of women living in the Estrie Region of Quebec, Canada. J Ovarian Res.

[12] Brinton LA, Lamb EJ, Moghissi KS, Scoccia B, Althuis MD (2004). Ovarian cancer risk associated with varying causes of infertility. Fertil Steril.

[13] Giudice LC, Kao LC (2004). Endometriosis. Lancet.

[14] Buis CC, van Leeuwen FE, Mooij TM, Burger CW, OMEGA Project Group (2013). Increased risk for ovarian cancer and borderline ovarian tumours in subfertile women with endometriosis. Hum Reprod.

[15] Siegel R, Naishadham D, Jemal A (2013). Cancer statistics, 2013. CA Cancer J Clin.

[16] Cao Q, Lu K, Dai S, Hu Y, Fan W (2014). Clinicopathological and prognostic implications of the miR-200 family in patients with epithelial ovarian cancer. Int J Clin Exp Pathol.

[17] Li Y, Zhang Z (2014). Potential microRNA-mediated oncogenic intercellular communication revealed by pan-cancer analysis. Sci Rep.

[18] Xiong M, Jiang L, Zhou Y, Qiu W, Fang L (2012). The miR-200 family regulates TGF-β1-induced renal tubular epithelial to mesenchymal transition through Smad pathway by targeting ZEB1 and ZEB2 expression. Am J Physiol Renal Physiol.

[19] Davidson B, Tropé CG, Reich R (2012). Epithelial-mesenchymal transition in ovarian carcinoma. Front Oncol.

[20] Vilming Elgaaen B, Olstad OK, Haug KB, Brusletto B, Sandvik L (2014). Global miRNA expression analysis of serous and clear cell ovarian carcinomas identifies differentially expressed miRNAs including miR-200c-3p as a prognostic marker. BMC Cancer.

[21] Zhao Q, Zhang B, Shao Y, Chen L, Wang X (2014). Correlation between the expression levels of miR-1 and PIK3CA in non-small-cell lung cancer and their relationship with clinical characteristics and prognosis. Future Oncol.

[22] Yu QQ, Wu H, Huang X, Shen H, Shu YQ (2014). MiR-1 targets PIK3CA and inhibits tumorigenic properties of A549 cells. Biomed Pharmacother.

[23] Stahlhut C, Suárez Y, Lu J, Mishima Y, Giraldez AJ (2012). miR-1 and miR-206 regulate angiogenesis by modulating VegfA expression in zebrafish. Development.

[24] Bronisz A, Wang Y, Nowicki MO, Peruzzi P, Ansari KI (2014). Extracellular vesicles modulate the glioblastoma microenvironment via a tumor suppression signaling network directed by miR-1. Cancer Res.

[25] Pan X, Wang R, Wang ZX (2013). The potential role of miR-451 in cancer diagnosis, prognosis, and therapy. Mol Cancer Ther.

[26] Bandres E, Bitarte N, Arias F, Agorreta J, Fortes P (2009). microRNA-451 regulates macrophage migration inhibitory factor production and proliferation of gastrointestinal cancer cells. Clin Cancer Res.

[27] Wang R, Wang ZX, Yang JS, Pan X, De W (2011). MicroRNA-451 functions as a tumor suppressor in human non-small cell lung cancer by targeting ras-related protein 14 (RAB14). Oncogene.

[28] Godlewski J, Bronisz A, Nowicki MO, Chiocca EA, Lawler S (2010). microRNA-451: A conditional switch controlling glioma cell proliferation and migration. Cell Cycle.

[29] Tian Y, Nan Y, Han L, Zhang A, Wang G (2012). MicroRNA miR-451 downregulates the PI3K/AKT pathway through CAB39 in human glioma. Int J Oncol.

[30] Dong Y, Zhao J, Wu CW, Zhang L, Liu X (2013). Tumor suppressor functions of miR-133a in colorectal cancer. Mol Cancer Res.

[31] Wang H, An H, Wang B, Liao Q, Li W (2013). miR-133a represses tumour growth and metastasis in colorectal cancer by targeting LIM and SH3 protein 1 and inhibiting the MAPK pathway. Eur J Cancer.

[32] Wang L, Li X, Zhou Y, Shi H, Xu C (2014). Downregulation of miR-133 via MAPK/ERK signaling pathway involved in nicotine-induced cardiomyocyte apoptosis. Naunyn Schmiedebergs Arch Pharmacol.

[33] Guo J, Xia B, Meng F, Lou G (2014). miR-133a suppresses ovarian cancer cell proliferation by directly targeting insulin-like growth factor 1 receptor. Tumour Biol.

[34] Annunziata CM, Stavnes HT, Kleinberg L, Berner A, Hernandez LF (2010). Nuclear factor kappaB transcription factors are coexpressed and convey a poor outcome in ovarian cancer. Cancer.

[35] Dinulescu DM, Ince TA, Quade BJ, Shafer SA, Crowley D (2005). Role of K-ras and Pten in the development of mouse models of endometriosis and endometrioid ovarian cancer. Nat Med.

